# Proximity screening greatly enhances electronic quality of graphene

**DOI:** 10.1038/s41586-025-09386-0

**Published:** 2025-08-20

**Authors:** Daniil Domaretskiy, Zefei Wu, Van Huy Nguyen, Ned Hayward, Ian Babich, Xiao Li, Ekaterina Nguyen, Julien Barrier, Kornelia Indykiewicz, Wendong Wang, Roman V. Gorbachev, Na Xin, Kenji Watanabe, Takashi Taniguchi, Lee Hague, Vladimir I. Fal’ko, Irina V. Grigorieva, Leonid A. Ponomarenko, Alexey I. Berdyugin, Andre K. Geim

**Affiliations:** 1 Department of Physics and Astronomy, University of Manchester, Manchester, UK; 2 National Graphene Institute, University of Manchester, Manchester, UK; 3 Department of Materials Science and Engineering, National University of Singapore, Singapore, Singapore; 4 Department of Physics, National University of Singapore, Singapore, Singapore; 5 National Institute for Materials Science, Tsukuba, Japan; 6 Department of Physics, University of Lancaster, Lancaster, UK

**Keywords:** Electronic properties and materials, Electronic properties and devices

## Abstract

The electronic quality of two-dimensional systems is crucial when exploring quantum transport phenomena. In semiconductor heterostructures, decades of optimization have yielded record-quality two-dimensional gases with transport and quantum mobilities reaching close to 10^8^ and 10^6^ cm^2^ V^−1^ s^−1^, respectively^1–10^. Although the quality of graphene devices has also been improving, it remains comparatively lower^11–17^. Here we report a transformative improvement in the electronic quality of graphene by employing graphite gates placed in its immediate proximity, at 1 nm separation. The resulting screening reduces charge inhomogeneity by two orders of magnitude, bringing it down to a few 10^7^ cm^−2^ and limiting potential fluctuations to less than 1 meV. Quantum mobilities reach 10^7^ cm^2^ V^−1^ s^−1^, surpassing those in the highest-quality semiconductor heterostructures by an order of magnitude, and the transport mobilities match their record^9,10^. This quality enables Shubnikov–de Haas oscillations in fields as low as 1 mT and quantum Hall plateaux below 5 mT. Although proximity screening predictably suppresses electron–electron interactions, fractional quantum Hall states remain observable with their energy gaps reduced only by a factor of 3–5 compared with unscreened devices, demonstrating that many-body phenomena at spatial scales shorter than 10 nm remain robust. Our results offer a reliable route to improving electronic quality in graphene and other two-dimensional systems, which should facilitate the exploration of new physics previously obscured by disorder.

## Abstract (web summary)

Screening by a graphite gate placed at 1 nm proximity to graphene produces transformative improvement in its electronic quality, reducing charge inhomogeneity by two orders of magnitude.

## Main

Among two-dimensional systems, those based on GaAlAs heterostructures have continuously maintained the record for electronic quality^1–10^. Their latest milestone—transport mobilities *µ* up to 5.7 × 10^7^ cm^2^ V^−1^ s^−1^ at subkelvin temperatures (*T*) and electron densities of approximately 1.5 × 10^11^ cm^−2^—came after three decades of painstaking work that yielded a fivefold improvement in mobility^9,10^. Graphene, despite being a relatively recent addition to two-dimensional systems, has also advanced in three big leaps. Starting from *µ* ≈ 10^4^ cm^2^ V^−1^ s^−1^ in early devices on oxidized Si wafers^11^, its mobility first rose to approximately 10^5^ cm^2^ V^−1^ s^−1^ and then to approximately 10^6^ cm^2^ V^−1^ s^−1^, which was demonstrated for graphene both suspended^12,13^ and encapsulated in hexagonal boron nitride (hBN)^14–17^. These mobilities have been observed for carrier densities *n* ≈ 10^10^–10^12^ cm^−2^ at liquid-helium *T*. Although graphene holds the room-*T* mobility record among all known materials (*µ* exceeds 0.15 × 10^6^ cm^2^ V^−1^ s^−1^ at *n* ≈ 10^11^ cm^−2^, limited by phonon scattering)^16–18^, its mobility at low *T* is, at present, constrained by two extrinsic factors: edge scattering and charge inhomogeneity. Indeed, hBN-encapsulated graphene devices are typically less than 10 µm in size, making edge scattering dominant at high densities, whereas the charge inhomogeneity δ*n* (exceeding a few 10^9^ cm^−2^ in the best-quality devices) limits transport at low *n*. A recent study^17^ using devices up to 40 µm wide showed that charged impurities also restricted *µ* at high *n* to a few 10^6^ cm^2^ V^−1^ s^−1^. Another key measure of electronic quality is quantum mobility *µ*_q_, which determines the onset of Landau quantization and related phenomena, such as Shubnikov–de Haas (SdH) oscillations and the quantum Hall effect (QHE). The highest-quality semiconductor heterostructures exhibit SdH oscillations in magnetic fields *B* down to approximately 10 mT (refs. ^9,19,20^), which translates into *µ*_q_ ≈ 10^6^ cm^2^ V^−1^ s^−1^. State-of-the-art graphene devices require several times higher *B* if we are to observe SdH oscillations. Below we show that proximity gates^21^ enable an unprecedented level of quality: they allow *µ*_q_ ≈ 10^7^ cm^2^ V^−1^ s^−1^ and reduce inhomogeneity to 3 × 10^7^ cm^−2^, that is, approximately 100 times lower than in the best devices with remote graphite gates and equivalent to one charge carrier left at the neutrality point per few micrometres squared. At low *n*, where edge scattering does not limit the mean free path *ℓ*, transport mobilities exceed  10^8^ cm^2^ V^−1^ s^−1^.

## Proximity-gated devices

**Fig. 1:**
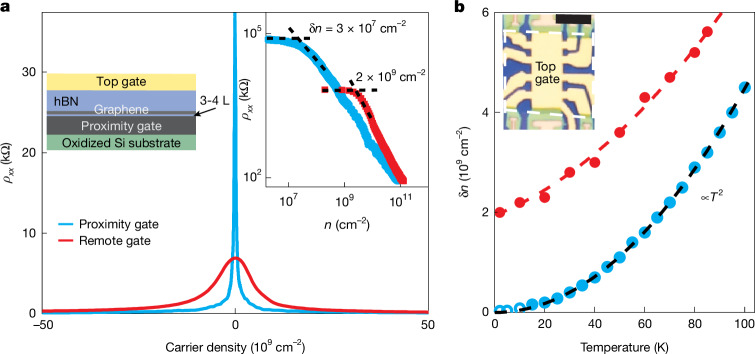
Profound effect of proximity screening on charge homogeneity. **a**, *ρ*_*xx*_(*n*) characteristic of state-of-the-art devices with remote graphite gates (red curve) and our proximity-gated devices (blue, device S1); *B* = 0 and *T* ≈ 2 K. Although the curves might look like many curves in the literature, the blue one is approximately 100 times narrower than for any previously reported device. The blue curve reaches approximately 100 kΩ but is cut off for clarity. Left inset, schematic of proximity-gated devices. Right inset, illustrates how δ*n* was evaluated. **b**, Temperature dependence of δ*n* for devices with proximity and remote gates (colour-coding as in **a**). The black parabola is δ*n* expected for perfect graphene. The red curve is the expected combined effect of residual inhomogeneity and thermal excitations^18^. Open blue circles indicate the low-*T* regime affected by the metal–insulator transition discussed in the Methods. Inset, optical micrograph of one of our proximity-gated devices. White dashed lines outline the graphite gate underneath. Scale bar, 10 µm.

Our devices were double-gated multi-terminal Hall bars fabricated from graphene monolayers sandwiched between two hBN crystals. The top hBN (20–70 nm thick) served as a gate dielectric for an evaporated Au/Cr electrode. A graphite crystal was used as the bottom gate (left inset of Fig. 1a; for details of fabrication, see Methods and Supplementary Information). A distinguishing feature of our devices is the ultrathin bottom hBN (3–4 atomic layers). Such a small thickness *d* ≈ 1 nm was chosen to reduce the background electrostatic potential through image-charge screening, thereby suppressing electron–hole puddles and scattering. Indeed, in the presence of a metal gate, the background potential should be reduced proportionally to [1 − exp(−2π*αd*/${\mathcal{L}}$)], where ${\mathcal{L}}$ is the characteristic size of the external potential variations and *α* ≈ 1.5 accounts for the anisotropy of the dielectric constant of hBN^21^. Given the large value of 2π*α*, potential variations larger in size than approximately 10 nm should be strongly suppressed for *d* = 1 nm. We could not employ thinner hBN because quantum tunnelling causes considerable electrical leakage. Even with trilayer hBN, to avoid leakage when varying *n*, we could only use the top gate. The use of atomically flat graphite crystals as proximity gates was also essential, as it prevented interfacial charges from becoming trapped and electric-field fluctuations caused by surface roughness (‘Device fabrication’ in Methods). The applied gate voltage was converted into *n* using Hall measurements, which was essential because of the large contribution from quantum capacitance for small *d* (‘Converting gate voltages into carrier density’ in Methods). The main challenge in fabricating the described devices was to obtain sufficiently large (several hundred micrometres squared), few-layer hBN crystals, which limited the width *W* of our Hall bars and, consequently, enhanced the role of edge scattering. We studied seven such devices with *W* from 6 to 10 µm (inset of Fig. 1b). For comparison, reference devices were also made using the same procedures but with remote graphite gates (*d* ≳ 20 nm) and standard Si-wafer gates.

## Extraordinary homogeneity

The impact of proximity screening on electronic quality is evident from Fig. 1a, which compares the longitudinal resistivity *ρ*_*xx*_ measured for devices with proximity and remote graphite gates. The reference device exhibited *µ* ≈ 10^6^ cm^2^ V^−1^ s^−1^, and the peak in *ρ*_*xx*_(*n*) was narrower than 10^10^ cm^−2^, characteristics rarely achievable without the use of graphite gates. Notably, our proximity-gated devices exhibited much sharper peaks (Fig. 1a), indicating even higher mobility and better homogeneity. This observation qualitatively agrees with the previous reports that found that placing graphene monolayers directly on graphite or graphene improved the optical and tunnelling spectra^22,23^. To quantify charge homogeneity in our devices, we used the standard approach^24,25^ and plot *ρ*_*xx*_(*n*) on a log–log scale (inset of Fig. 1a). At low *T*, graphene starts responding to gate doping only above a characteristic carrier density δ*n* because of electron–hole puddles. Although the reference device exhibited δ*n* ≈ 2 × 10^9^ cm^−2^ (Fig. 1 and Extended Data Fig. 2), matching the lowest δ*n* reported in the literature, proximity screening reduced δ*n* by as much as 100 times (Fig. 1 and Extended Data Fig. 3). With increasing *T*, δ*n* increased (Fig. 1b) because of thermally excited electrons and holes, which appeared at the neutrality point in densities *n*_th_ = (π/6)(*k*_B_*T*)^2^/(*ħv*_F_)^2^, where *k*_B_ and *ħ* are the Boltzmann and reduced Planck constants and *v*_F_ is the Fermi velocity of graphene. The extra carriers broadened the *ρ*_*xx*_(*n*) peak, with their contribution described^18,25^ by δ*n* ≈ *n*_th_/2, in agreement with our measurements on proximity-gated devices (Fig. 1b and Extended Data Fig. 3b). For reference devices, residual inhomogeneity meant that δ*n* converged with the theoretical dependence only above 150 K (Extended Data Fig. 2c).

Note that, below 10 K, proximity-gated devices often exhibit a logarithmic-kind increase in *ρ*_*xx*_(*T*) at the neutrality point, with values reaching well above the resistance quantum *h*/*e*^2^, indicating the emergence of a low-*T* insulating state that could be destroyed by small *B* ≈ 1 mT (‘Metal–insulator transition’ in Methods). As this insulating state may have affected our evaluation of δ*n* below 10 K if we were using the standard procedure, we verified the values obtained in the low-*T* regime using measurements of *ρ*_*xy*_(*n*). The Hall resistivity switched sharply between electron and hole doping (‘Charge inhomogeneity from Hall measurements’ in Methods), and the width of the switching region provides an alternative quantitative measure of charge inhomogeneity^18^, whereas *ρ*_*xy*_ is expected to be less sensitive to localization effects. We found close agreement between δ*n* extracted using both Hall and longitudinal resistivities over the entire *T* range, corroborating the threefold decrease in δ*n* between 2 and 10 K (Fig. 1b).

## Transport mobilities

**Fig. 2:**
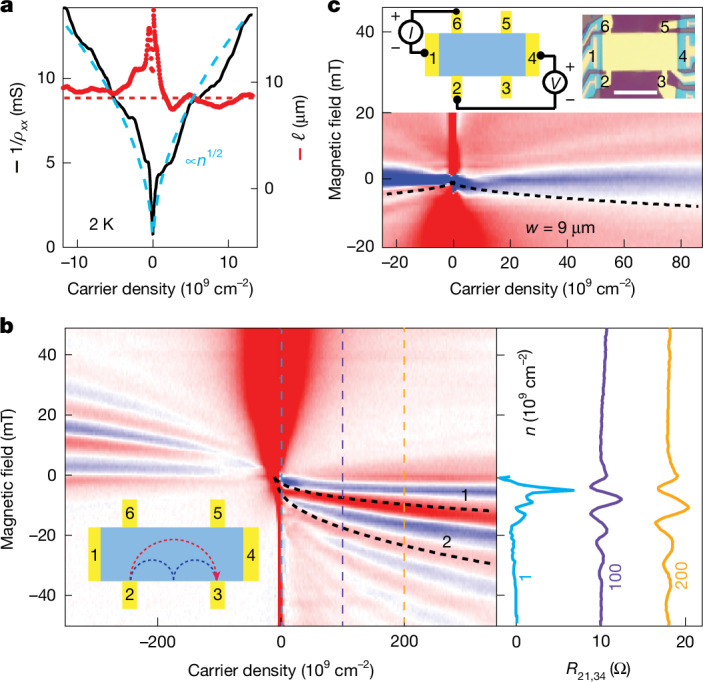
Ballistic transport in proximity-screened graphene. **a**, Conductivity and mean free path (black and red curves, respectively). The blue dashed curve shows the *n*^1/2^ dependence expected for transport limited by edge scattering (the best fit yields *ℓ* ≈ 9 µm). The red line indicates the actual device width of approximately 8.5 µm. **b**, Ballistic transport probed by magnetic focusing. Left, map of focusing resistance *R*_21,34_(*n*, *B*) = *V*_34_/*I*_21_ (blue-to-red scale, −5 Ω to 5 Ω). The current *I*_21_ is driven between contacts 2 and 1 as shown in the inset. The voltage *V*_34_ is measured between 3 and 4. *L* ≈ 13.5 µm. Black dashed curves are the expected positions of the first two focusing peaks (corresponding trajectories shown in the inset). Right, vertical cuts at fixed *n* marked in the map by colour-coded dashed lines. **c**, Example of bend-resistance measurements. Insets, measurement geometry (left) and an optical micrograph of the device (right). Colour map shows *R*_61,42_ (colour scale as in **b**). The dashed curve is *W* = *D*_c_/2, the condition where the bend resistance is expected to reverse its sign^15,26^. Data in **a** are for device S4 at 2 K. Data in **b** and **c** are for device S6 (at 20 K to suppress mesoscopics). Scale bar, 10 µm.

To evaluate *µ*, we first employed the standard expression *µ* = 1/*neρ*_*xx*_. Its use necessitates a cutoff at a finite *n* of a few δ*n*, where electron–hole puddles start contributing to transport^18^. For the reference devices, this means that the cutoff was at approximately 10^10^ cm^−2^ and it placed a limit on the maximum achievable mobility as *µ* = (*eℓ*/*ħ*)(π*n*)^−1/2^ ≲ 10^7^ cm^2^ V^−1^ s^−1^, assuming edge scattering dominates (*ℓ* ≈ *W* ≈ 10 µm). At these *n*, our limited-size proximity-gated devices could also not exceed this limit. However, their exceptional homogeneity pushed the cutoff to much lower *n*. Figure 2a shows that *ρ*_*xx*_ continues to scale approximately as *n*^−1/2^ into the densities below 10^10^ cm^−2^. For *n* ≳ 3–5 × 10^9^ cm^−2^, the extracted *ℓ* closely matches *W* (red curve in Fig. 2a), as expected for ballistic transport. This also yields *µ* ≈ 10^7^ cm^2^ V^−1^ s^−1^. For even lower *n*, but well outside the electron–hole puddle regime emerging below 10^8^ cm^−2^, the mobility evaluated from *ρ*_*xx*_(*n*) continued to increase with decreasing *n*, reaching above 2.5 × 10^7^ cm^2^ V^−1^ s^−1^ at *n* ≈ 10^9^ cm^−2^, a very high value for graphene. However, at such low *n*, the standard approach for evaluating *µ* can be questioned because the extracted *ℓ* are comparable to or start exceeding *W* (Fig. 2a). Although specular reflection at graphene edges can lead to *ℓ* > *W* (feasible because the Fermi wavelength *λ*_F_ exceeds 1 µm), the estimate may be skewed by interference (‘mesoscopic’) fluctuations (Fig. 2a and Methods).

As an alternative means of determining *ℓ* and, therefore, *μ*, we employed measurement geometries that can probe ballistic transport directly. One of them is magnetic focusing, in which charge carriers injected from one contact are collected by another contact at distance *L* (inset of Fig. 2b). A magnetic field bends the trajectories, creating caustics that lead to focusing resonances^26,27^. We found that their positions accurately matched those expected theoretically (Fig. 2b and Extended Data Fig. 5). Importantly, the focusing resonances in proximity-gated devices survived down to *n* ≈ 10^9^ cm^−2^ (Fig. 2b), whereas in our best reference device with *µ* ≈ 7 × 10^6^ cm^2^ V^−1^ s^−1^, magnetic focusing could be observed only above 10^11^ cm^−2^, and even higher *n* were required for Si-gated devices (Extended Data Fig. 5). For the proximity-gated device in Fig. 2b, the observation of magneto-focusing peaks implies that charge carriers have *ℓ* ≳ π*D*_c_/2 = π*L*/2 ≈ 21 µm (*D*_c_ is the cyclotron diameter). Using the semi-quantitative criterion detailed in Methods (‘Magnetic focusing in reference devices’), we found that the persistence of focusing peaks down to 10^9^ cm^−2^ yields *µ* ≳ 6 × 10^7^ cm^2^ V^−1^ s^−1^, consistent with our above estimates from the *ρ*_*xx*_(*n*) dependence.

## Ballistic Dirac plasma

Next, we employed the bend-resistance geometry^15,26^ (Fig. 2c). In zero *B*, the measured bend resistance remained negative at all *n*, showing that carriers travelled ballistically between the injector and collector (*ℓ* > *W* ≈ 9 µm). Even at the neutrality point, where Dirac fermions formed a Boltzmann rather than Fermi gas and experienced frequent Planckian scattering^25^ (‘Metal–insulator transition’ in Methods), the transport in our proximity-gated devices remained ballistic. The carrier density in the Dirac plasma is given by *n*_th_ and was approximately 3 × 10^8^ cm^−2^ for the 20-K measurements in Fig. 2c. This yields *µ* = (*eℓ*/*ħ*)(π*n*)^−1/2^ ≥ 5 × 10^7^ cm^2^ V^−1^ s^−1^, in close agreement with the *µ* found above in the Fermi regime. The negative bend resistance at the neutrality point was observed down to 10 K, indicating that, in the low-*T* Dirac plasma, *µ* could exceed 10^8^ cm^2^ V^−1^ s^−1^. However, interference fluctuations (*λ*_F_ ≳ 3 µm) emerging at these *T* started criss-crossing the colour map near zero *n*, making the last estimate less reliable. Independent evidence for ballistic transport in the Dirac plasma at *T* close to 10 K comes from the *ρ*_*xx*_(*T*) dependence at the neutrality point (Extended Data Fig. 4). Above 50 K, proximity-gated devices exhibited the expected constant resistivity of approximately 1 kΩ, characteristic of Planckian scattering^25,28^. At lower *T* but before the onset of the insulating state at approximately 10 K, *ρ*_*xx*_ was found to evolve as 1/*T* (Extended Data Fig. 4c). The *T* dependence yields scattering times shorter than the Planckian limit and given by *W*/*v*_F_ (‘Metal–insulator transition’ in Methods), thus confirming ballistic transport limited by edge scattering down to 10 K. This supports our above estimate for *µ* ≳ 10^8^ cm^2^ V^−1^ s^−1^ in the low-*T* Dirac plasma of proximity-gated devices.

A direct comparison of graphene with other two-dimensional systems in terms of their transport mobilities was unfortunately hindered by the very different density ranges where ballistic transport is observed. The record mobilities for GaAlAs heterostructures were achieved in a narrow range of *n* ≈ 1–2 × 10^11^ cm^−2^, whereas, at such densities, the mobility even in our reference devices was limited by their *W*. Additionally, the scattering mechanisms fundamentally differed between the two systems. Transport in high-quality hBN-encapsulated graphene is primarily limited by edge scattering and background fluctuations (‘Sources of disorder’ in Methods), whereas mobilities in GaAlAs heterostructures are limited by ionized impurities^6,8,9^. The different electronic spectra (linear versus parabolic dispersion) also affect the scattering efficiency. Nonetheless, as *µ* generally increases with *n* due to improved self-screening^17^, even higher mobilities are probably achievable in graphene at the densities typical of GaAlAs heterostructures, if larger proximity-gated devices become available to reduce edge scattering.

## High quantum mobilities

**Fig. 3:**
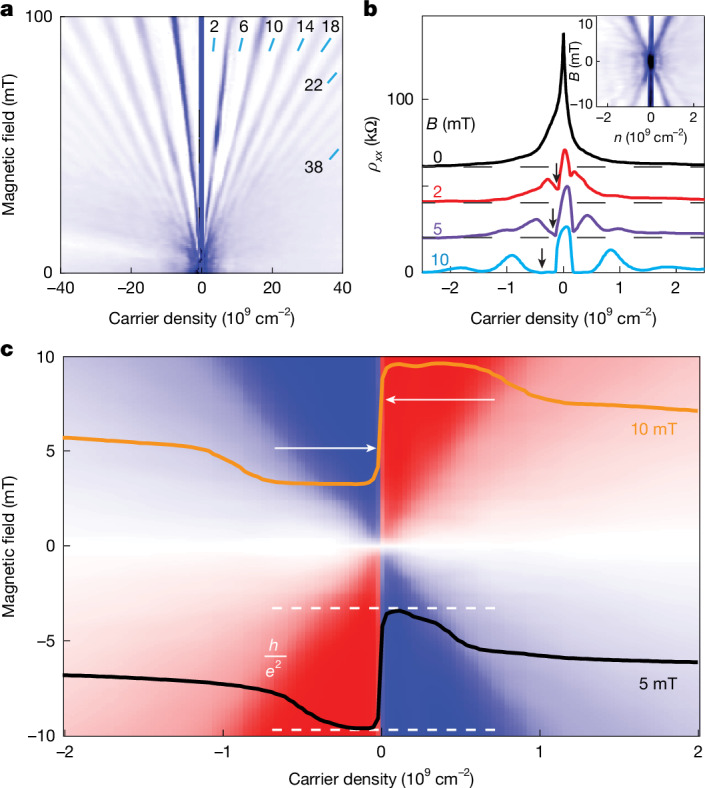
Quantization in millitesla fields. **a**, Landau fan diagram *ρ*_*xx*_(*n*,*B*) (white-to-blue scale, 0 to 4 kΩ). Numbers with blue dashes denote *ν*. **b**, Horizontal cuts from **a** at different *B*. Inset, details of the fan diagram in low *B* (white-to-blue, 0 to 40 kΩ). Arrows: expected positions for *ν* = −2. Note that *ρ*_*xx*_(*n*) changes rapidly near the neutrality point, which results in a wide dark region that obscures the onset of SdH oscillations. They are better resolved on the horizontal cuts (also, see Extended Data Fig. 6). **c**, Map for *ρ*_*xy*_ (blue-to-red scale, ±*h*/2*e*^2^). The superimposed curves show *ρ*_*xy*_(*n*) traces at 5 mT and 10 mT (offset for clarity). Arrows mark the full transition width at half-height, approximately 6 × 10^7^ cm^−2^. All data are for device S1 at 2 K.

A more direct comparison between graphene and other two-dimensional systems was made using quantum mobility, which is insensitive to device size and geometry. Our proximity-gated devices demonstrated Landau quantization in strikingly low *B* (Fig. 3a). For example, at 50 mT and 2 K, we observed more than 15 SdH oscillations (filling factor *ν* > 60; cyclotron gaps between neighbouring levels down to 1.1 meV). This indicates that there were extremely narrow Landau levels, in excellent agreement with the measured δ*n*, that yielded spatial variations of the Dirac point of *ħv*_F_(πδ*n*)^1/2^ ≈ 0.5 meV. Furthermore, the SdH oscillations became apparent at the onset field B* ≈ 1–2 mT (Fig. 3b and Extended Data Fig. 6), much lower than B* in reference devices with remote graphite and Si-wafer gates (approximately more than 35 and 80 mT, respectively). To ensure that the observed minima in *ρ*_*xx*_(*n*) originated from Landau quantization rather than interference fluctuations, we not only checked their positions in the *n*–*B* parameter space (Fig. 3b) but also shifted mesoscopic features by cycling up to room *T* and varying the electric field across the graphene using both the top and bottom gates. These procedures altered both the rapidly rising background near the neutrality point and the positions of the fluctuations but left the SdH oscillations unaffected, confirming that the 1-mT minima were, indeed, due to Landau quantization. We believe that SdH oscillations in our proximity-gated devices appeared at even lower *B* but were obscured until their amplitude became comparable to *ρ*_*xx*_ (background subtraction revealed features down to approximately 0.5 mT). Note that, with a constant background, SdH oscillations typically become visible at amplitudes of just several per cent of *ρ*_*xx*_ (‘Onset of SdH oscillations’ in Methods). The use of higher *T* to suppress mesoscopic fluctuations was unpractical as it smeared the cyclotron gaps (1.2 meV for *ν* = ±2 at 1 mT), whereas reducing *T* below 2 K amplified the fluctuations (Extended Data Fig. 4a).

Quantum mobility can be estimated from the onset of SdH oscillations using expression *µ*_q_B* ≈ 1. Although this criterion is widely used in the literature, we validated its quantitative accuracy through studying SdH oscillations as a function of *B* (Extended Data Fig. 7). Their amplitude followed the Dingle formula exp(–π/*µ*_q_*B*), and we found *µ*_q_B* = 1 to hold within 10–20%, if the first discernible oscillation was considered as B* (‘Determination of quantum mobility’ in Methods). Thus, the Landau quantization visible on our plots at *B* = 1 mT yields *µ*_q_ of at least 10^7^ cm^2^ V^−1^ s^−1^, setting a lower bound for quantum mobility in proximity-gated devices. (As discussed in Methods, magnetic fields larger than the actual B* were required to resolve oscillations against the rising background and mesoscopics). As Landau quantization involves completed cyclotron orbits, this implies *ℓ* ≥ π*D*_c_, even if small-angle scattering (which disrupts the quantization but has a lesser effect on transport) is ignored. Using this *ℓ* in the expression for transport mobility gives *µ* ≥ 2π*µ*_q_ > 6 × 10^7^ cm^2^ V^−1^ s^−1^, consistent with our estimates from *ρ*_*xx*_(*n*).

In line with the early onset of SdH oscillations, the QHE fully developed at *B* ≤ 5 mT (Fig. 3c and Extended Data Fig. 8a). The transition between electron and hole plateaux at the neutrality point had a half-width at half-height of approximately 3 × 10^7^ cm^−2^ (Fig. 3c), providing an independent measure of charge inhomogeneity in proximity-gated devices. Importantly, the emergence of QHE plateaux was not constrained by the quality of the graphene but depended on the width *w* of the voltage probes, with plateaux developing earlier in devices with larger *w* (Extended Data Fig. 8b). This is attributed to the fact that, at such low *B*, both *D*_c_ ≈ 0.7 µm at 5 mT and the magnetic length *ℓ*_B_ = *D*_c_(*ν*/8)^1/2^ were comparable to *w* ≤ 1 µm, causing edge states to be partially reflected from the contacts and preventing the development of quantized plateaux^29^.

## Impact on many-body phenomena

**Fig. 4:**
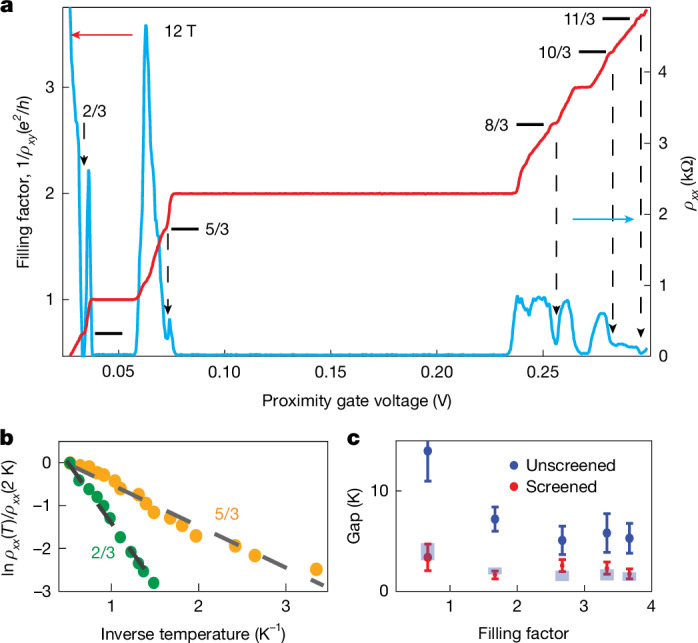
Fractional QHE in proximity-gated devices. **a**, *ρ*_*xy*_ and *ρ*_*xx*_ at 12 T and 50 mK (red and blue curves; left and right axes, respectively). Data are plotted as a function of proximity gate voltage, as an accurate conversion to the carrier density was unfeasible due to the 2.5-dimensional QHE in the graphite gate^36^ and negative quantum capacitance^31^. *ρ*_*xy*_ is plotted in terms of *ν* = (*h*/*e*^2^)/*ρ*_*xy*_. Horizontal lines mark expected positions of fractional plateaux. Arrows indicate the corresponding *ρ*_*xx*_ minima. **b**, Arrhenius plots for resistance minima (normalized by values at 2 K) at *ν* = 2/3 and 5/3 were used to extract the activation energies. **c**, Comparison of fractional QHE gaps in proximity-gated device S1 (red symbols) and a remote-gate device (blue symbols with error bars). Blue rectangular symbols are the expected gaps after proximity screening, calculated using the *ℓ*_B_/2*d* suppression factor with *d* = 1 nm and *ℓ*_B_ ≈ 7.5 nm for 12 T.

The achieved electronic quality comes with a trade-off. Many-body phenomena, which are of considerable interest and tend to emerge with each order-of-magnitude improvement in quality, are unavoidably suppressed by enhanced screening^21,30^. To assess how proximity screening affects many-body phenomena in graphene, we studied the fractional QHE in our devices (*B* up to 12 T and *T* down to 50 mK). Figure 4a shows distinct Hall plateaux and the corresponding resistivity minima at *ν* = 2/3, 5/3, 8/3, 10/3 and 11/3. Notably, no signatures of the 1/3 state could be observed, despite it usually being most profound. We attribute its absence to negative quantum capacitance, which can suppress this state in gate voltage measurements^31^ (‘Fractional QHE under proximity screening’ in Methods). We extracted fractional QHE energy gaps (Fig. 4b) and compared them with those in our reference devices, which exhibited gaps consistent with those reported in the literature^32–34^ (Fig. 4c). Although proximity screening reduced the fractional gaps by a factor of 3–5 compared to unscreened devices, the gaps remained substantial, larger than those typical of other two-dimensional systems.

The suppression factor can be understood as follows. The interaction energy responsible for the fractional QHE scales as *e*^2^/*εℓ*_B_ (two electrons at distance *ℓ*_B_ signifying the spatial scale of electron–electron interactions in a magnetic field, and *ε* is the effective dielectric constant), whereas proximity screening changes this expression to *e*^2^2*d*/*εℓ*_B_^2^ (two interacting dipoles each with an electron and its image charge at distance 2*d*)^30^. This yields a suppression factor of *ℓ*_B_/2*d* ≈ 4 at 12 T, in good agreement with our observations (Fig. 4c). The fractional states first appeared above 7 T and became more pronounced at higher fields where *ℓ*_B_ decreased below 10 nm. This length scale matches the spatial range over which the proximity screening with *d* ≈ 1 nm suppressed background electrostatic potential because of the discussed large factor 2π*α*. The same 10-nm scale was previously noticed in the suppression of two other types of interaction phenomena (electron viscosity and Umklapp scattering)^21^. These observations indicate that, although proximity screening does reduce the interaction strengths, many-body physics involving scales shorter than 10 nm should remain accessible in proximity-gated devices.

## Conclusion

Our study demonstrates that proximity screening can improve the electronic quality of graphene by up to two orders of magnitude. The resulting charge homogeneity is unprecedented (Dirac point fluctuations of less than 10 K), enabling extremely narrow Landau levels and the QHE in a few millitesla. Although the quality achieved comes at the expense of suppressing many-body phenomena, interactions involving relatively short spatial scales (less than 10 nm) remained strong, indicating that proximity screening may be particularly valuable for studying short-range correlated states and many-body physics in high magnetic fields. We anticipate that the approach will prove especially beneficial for studying graphene multilayers and superlattices. As the quality of two-dimensional semiconductors continues to improve, proximity screening may also be relevant for these systems where richer band structures and stronger interactions than in monolayer graphene may reveal new physics enabled by reduced disorder. Alternatively, our approach could be used to intentionally suppress many-body interactions while simultaneously delivering superior electronic quality, as demonstrated by the observation of the helical QHE^35^ in our proximity-gated devices at fields below 80 mT (‘Helical quantum Hall transport’ in Methods).

## Methods

### Device fabrication

The devices were fabricated using the standard van der Waals assembly and electron-beam lithography, following procedures established in the literature without introducing critical modifications. Our detailed protocols are provided in Supplementary Information and summarized below.

Monolayer graphene, hBN crystals (both 30–70 nm thick and 3–4 layers thin) and relatively thick graphite (5–50 nm) were mechanically exfoliated onto an oxidized Si wafer (290 or 70 nm SiO_2_). Their thicknesses were determined using optical contrast, atomic force microscopy and Raman spectroscopy (Supplementary Information). For most of the reference devices with remote graphite and Si gates, we assembled the exfoliated crystals using polymer-free flexible silicon nitride membranes^17^. For all proximity-gated devices and some reference devices, we employed polydimethylsiloxane stamps with polypropylene carbonate as a sacrificial layer. Although not critical, we found the latter method preferable for proximity-gated devices as it imposes fewer constraints (Supplementary Information).

We began the assembly of encapsulated graphene by picking up a selected top hBN crystal and then using it to sequentially collect graphene and bottom hBN crystals. Crucially for achieving ultrahigh electronic quality, the stacking process required the slow deposition of two-dimensional crystals onto each other, as this minimized the formation of bubbles and wrinkles and, consequently, allowed the sufficiently large final devices (Supplementary Fig. 1). All assembly procedures were conducted in air. Exfoliated flakes were either used within an hour or stored in a glovebox for assembly within 2–3 weeks. The completed trilayer stacks were transferred onto either a graphite crystal (for graphite-gated devices) or an oxidized Si wafer.

Following the assembly, we used electron-beam lithography to define the top gate region and we deposited Cr–Au metallization by electron-beam evaporation. In the subsequent lithography step, we created electrical contacts by first exposing graphene edges using reactive-ion etching and then depositing Cr–Au to form one-dimensional contacts. The typical contact resistance was 2–5 kΩ µm at the neutrality point and decreased down to 0.1–0.3 kΩ µm at high doping. In the final step, we used the metallic top gate as an etching mask and defined multi-terminal Hall bars, as shown in the micrographs throughout the main text, Methods and Supplementary Information.

Although some devices failed during fabrication (typically because of poor one-dimensional contacts), all successfully fabricated proximity-gated devices demonstrated consistently ultrahigh quality, with SdH oscillations always emerging below 4–5 mT and sometimes below 1 mT. The primary determinants of our device quality were interface cleanliness and careful, slow stacking (as described above and detailed in Supplementary Information).

### Electrical measurements

Magnetotransport measurements were carried out in two cryogenic systems. A liquid-He cryostat with a variable temperature insert was used to study transport from 1.7 K up to room *T*. For measurements in the fractional QHE regime, we used a dilution refrigerator (Oxford Instruments) with *T* from 6 K down to 50 mK and magnetic fields up to 12 T.

For resistance measurements, we used the standard low-frequency lock-in technique at 30.5 Hz. Typically, the a.c. currents were between 1 and 5 nA, which allowed us to avoid heating and self-gating effects. Higher currents were required in magneto-focusing and bend-resistance experiments to achieve sufficient signal-to-noise ratios: up to 1 µA for devices with remote gates and up to 0.1 µA for proximity-gated devices.

To control the carrier density *n*, we applied gate voltages using a Keithley Sourcemeter-2636B. For proximity-gated devices, in most cases we used only the top Au gate while keeping the bottom graphite or Si gate grounded. This approach prevented tunnelling through and possible breakdown of the few-layer hBN dielectric between the graphene and the proximity gate. However, for measurements of the fractional QHE, we found it advantageous to use the proximity gate to vary *n*. This strategy allowed us to avoid insulating QHE states inside the graphite gate (due to the 2.5-dimensional QHE in graphite^36^). Simultaneously, we applied a large bias to the bottom Si gate to suppress insulating states in regions of the graphene electrical leads that were not covered by the graphite gate.

### Converting gate voltages into carrier density

**Extended Data Fig. 1:**
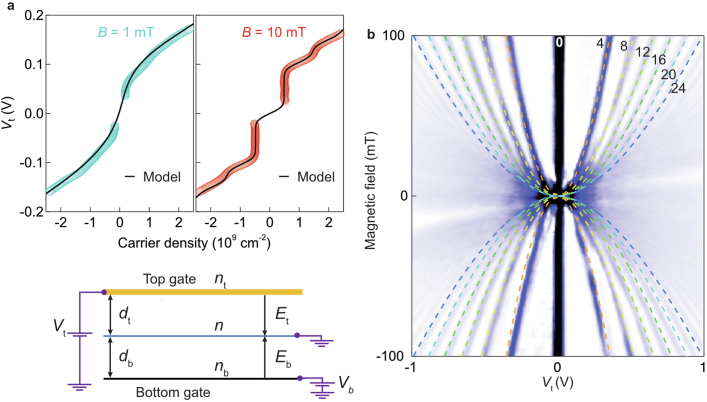
Converting gate voltages into carrier density. **a** Gate voltage *V*_t_ as a function of carrier density *n* determined from *ρ*_xy_ measurements at 1 and 10 mT (left and right panel, respectively). Symbols: Experimental data. Solid curves: Best fits using our quantum capacitance model with Landau level broadening *Γ* = 0.25 meV. Bottom panel: Device schematics. Applied voltages *V*_t_ and *V*_b_ create electric fields *E*_t_ and *E*_b_ in hBN layers (thicknesses *d*_t_ and *d*_b_). Charge densities *n*_t_ and *n*_b_ at the gates induce the carrier density *n* in graphene. **b** Landau fan diagram *ρ*_xx_(*V*_t_,*B*) measured at 2 K for proximity-gated device S1 (white-to-blue scale: 0 to 4 kΩ). Dashed curves: Calculated positions of peaks in *ρ*_xx_ for the labeled filling factors.

For devices with relatively thick gate dielectrics, the carrier density *n* in graphene exhibits a practically linear dependence on gate voltage *V*_g_ because the geometric capacitance dominates (approximately 12 nF cm^−^^2^ for our Si-gated devices). The linearity holds because typical shifts in the chemical potential of graphene are small compared with *eV*_g_. However, the simple relation *n* ∝ *V*_g_ breaks down notably in proximity-gated devices with their ultrathin dielectrics, as high-quality graphene exhibits a vanishingly small density of states near the neutrality point and in quantizing magnetic fields. This phenomenon is conventionally described in terms of quantum capacitance *C*_q_ = *e*∂*n/*∂*µ*, which contributes in series with the geometric capacitance and consequently dominates if the density of states and, hence, *C*_q_ become sufficiently small^37,38^. The resulting nonlinearities in the *n*(*V*_g_) dependence become particularly pronounced in ultrahigh-quality graphene, as elucidated by Extended Data Fig. 1. Below we describe our methodology for converting the gate voltage into a carrier density.

We modelled our devices as parallel-plate capacitors with top and bottom gates biased at *V*_t_ and *V*_b_ (bottom panel of Extended Data Fig. 1a). The gates were separated from the graphene by hBN layers of thicknesses *d*_b_ and *d*_t_. Applied voltages created electric fields *E*_t_ and *E*_b_ inside the top and bottom hBN dielectrics. *V*_t_ shifted the electrostatic potential by *d*_t_*E*_t_ and, therefore, changed the electrochemical potential of graphene *µ*(*n*), yielding the equation *eV*_t_ = *ed*_t_*E*_t_ + *µ*(*n*) where *e* denotes the absolute value of the electron charge. Similarly for the bottom gate, *eV*_b_ = *ed*_b_*E*_b_ + *µ*(*n*). The charge densities at the gate interfaces were *n*_t_ = −*εε*_0_*E*_t_/*e* and *n*_b_ = −*εε*_0_*E*_b_/*e* where *ε*_0_ is the vacuum permittivity and *ε* ≈ 3.5 is the hBN dielectric constant. Charge conservation requires *n*_t_ + *n*_b_ + *n* = 0, which yields the following equation connecting *n* and *µ* with the gate voltages:

1 $${V}_{{\rm{b}}}+\frac{{d}_{{\rm{b}}}}{{d}_{{\rm{t}}}}{V}_{{\rm{t}}}=\frac{e{d}_{{\rm{b}}}}{\varepsilon {\varepsilon }_{0}}n+\left(1+\frac{{d}_{{\rm{b}}}}{{d}_{{\rm{t}}}}\right)\frac{\mu (n)}{e}$$

This equation is generally applicable to any double-gated field-effect device that uses a two-dimensional material as a conducting channel. In most of our measurements, we used only the top gate to vary *n* while keeping both graphene and the proximity (bottom) gate grounded (*V*_b_ = 0). It is important to note that, according to the above equations, electric fields will still develop in the gate dielectrics, even when the graphene is electrically connected to one of the gates, provided the other gate shifts *µ*(*n*) from zero. Indeed, if *V*_b_ = 0, we obtain *E*_b_ = −*µ*(*n*)/*ed*_b_, which shows that, despite being grounded, the proximity gate with its ultrathin dielectric actively participates in the device electrostatics and, as *E*_b_ ∝ 1/*d*_b_, strongly contributes to the nonlinear behaviour of *n*(*V*_t_).

The electrochemical potential *µ*(*n*) in equation (1) can be calculated by numerically inverting the integral:

$$n(\mu )={\int }_{0}^{\infty }D(E)f(E,\mu ,T)\,{\rm{d}}E,$$

where *f*(*E*, *µ*, *T*) is the Fermi–Dirac distribution function. The density of states *D*(*E*) is modelled in graphene as

$$D(E)=\frac{4eB}{\sqrt{2{\rm{\pi }}}h\varGamma }\sum _{i}\exp \left(-\frac{{(E-{E}_{i})}^{2}}{2{\varGamma }^{2}}\right).$$

This equation is written for the general case of a finite magnetic field. Here, *E*_*i*_ is the energy of the *i*th Landau level, and *D*(*E*) depends not only on energy *E* but also on magnetic field *B* and level broadening *Γ* due to both temperature and disorder^37,38^. We solved equation (1) numerically (using an energy cutoff of 0.5 eV in all computations). Examples for non-quantizing and quantizing fields are illustrated in Extended Data Fig. 1a by black solid curves.

Experimentally, we determined the carrier density *n* as a function of *V*_t_ by measuring the Hall resistivity *ρ*_*xy*_(*V*_t_) at zero *V*_b_ and employing the standard expression *n* = *B*/*eρ*_*xy*_. The latter is valid only outside the mixed electron–hole regime near the neutrality point (see, for example, Extended Data Fig. 3a and ‘Charge inhomogeneity from Hall measurements’ in Methods), which resulted in gaps on our experimental curves for very low *n* ≲ δ*n* (Extended Data Fig. 1a). For the known thicknesses *d*_b_ and *d*_t_, we fitted our experimental data using the numerical model described above, with broadening *Γ* as the only fitting parameter. Extended Data Fig. 1a shows good agreement between the experimental *n*(*V*_t_) dependences and the model, yielding *Γ* ≈ 0.25 meV. This shows that, for the presented device, the energy broadening was dominated primarily by *T*. Our model accurately described the experimental curves in both non-quantizing and quantizing fields (left and right panels, respectively). We applied these fitted dependences (using the *Γ* values determined for each device) to convert *V*_*t*_ into *n* and present the experimental data for proximity-gated devices as a function of *n* for different *B* and *T*.

We assessed the accuracy of our conversion procedure by examining how well it describes the *ρ*_*xx*_ peak positions on the Landau fan diagram measured over a wide range of *V*_t_ and *B* (Extended Data Fig. 1b). For monolayer graphene in moderate *B*, these peaks should appear at filling factors *ν* = 0, ±4, ±8, ±12, … and follow the immutable relation *B*(*ν*) = *nh*/*eν* on the fan diagram. Using our model with the extracted *Γ* values, we calculated *n*(*V*_t_) and used this to determine the *B*(*V*_t_) dependences for different filling factors, as shown by dashed curves in Extended Data Fig. 1b. The excellent agreement between measured and predicted Landau-level positions confirms the accuracy of our procedure for converting gate voltages into carrier densities. Using the same procedure, we transformed the nonlinear fan diagrams obtained as functions of *V*_*t*_ (such as shown in Extended Data Fig. 1b) into the standard fan diagrams where Landau levels appear as straight lines radiating from the origin, as required by theory (Fig. 3 and Extended Data Fig. 6). These linear fan diagrams provide further confirmation of the accuracy of our approach.

### Charge inhomogeneity in the best remote-gated devices

**Extended Data Fig. 2:**
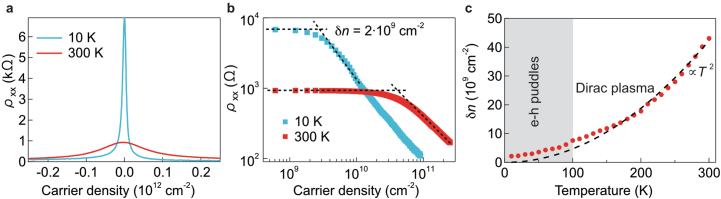
Transport in hBN-encapsulated graphene with remote graphite gates. **a** Zero-field *ρ*_xx_(*n*) at 10 and 300 K. **b** Same data on a log-log scale. Dashed lines show our evaluation of *δn*. **c** Temperature dependence of *δn*. Symbols: Experimental data. Black curve: Expected broadening due to thermally excited carriers. Grey region indicates that electron-hole puddles dominate. The region’s upper bound corresponds to *T* at which thermal broadening becomes twice that given by the puddles.

In the main text, we compared our proximity-gated devices with reference devices that used remote graphite gates. Extended Data Fig. 2 provides further details about our best remote-gated device. Its zero-*B* resistivity *ρ*_*xx*_(*n*) near the neutrality point is shown for two representative *T* (Extended Data Fig. 2a) and replotted on a log–log scale so that we could evaluate the residual charge inhomogeneity δ*n* (Extended Data Fig. 2b). With increasing *T*, the resistivity peak broadened because of thermally excited carriers. The extracted δ*n*(*T*) is shown in Extended Data Fig. 2c. Above 150 K, the experimental data follow the expected parabolic dependence δ*n* = *n*_th_(*T*)/2 ∝ *T*^2^ within our measurement accuracy, as discussed in the main text. Below 10 K, δ*n* saturates because electron–hole puddles dominate the transport properties.

### Charge inhomogeneity from Hall measurements

**Extended Data Fig. 3:**
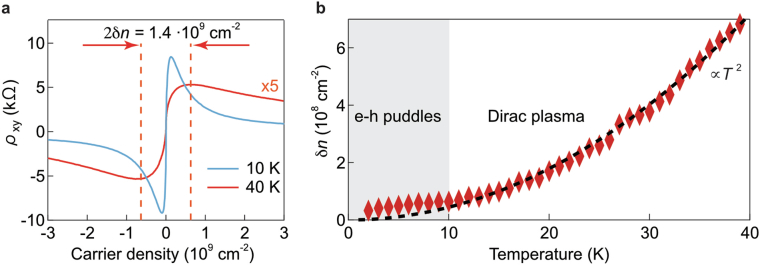
Estimating charge inhomogeneity using Hall resistivity. **a** *ρ*_xy_(*n*) near the NP at 10 and 40 K (color-coded curves). Measurements at 3 mT provided sufficiently large Hall voltages for high accuracy. The full width between the dashed lines corresponds to 2*δn* for the case of 40 K. **b** Temperature dependence of *δn* (symbols). Black dashed curves: ½*n*_th_(*T*). Grey region indicates the *T* range affected by electron-hole puddles.

An alternative way to evaluate the charge inhomogeneity δ*n* is to use Hall measurements^18^. Extended Data Fig. 3a shows *ρ*_xy_(*n*) for one of our proximity-gated devices at two representative *T*. The density range between the extrema in *ρ*_*xy*_(*n*) marks the regime of mixed electron–hole transport where the Hall response no longer follows the standard single-carrier dependence *ρ*_*xy*_(*n*) = *eB*/*n*. The distance between the extrema is given by *n*_th_ ≈ 2δ*n* and provides another quantitative measure of charge inhomogeneity^18^. On increasing *T* from 10 to 40 K, the extrema moved apart and broadened. Extended Data Fig. 3b compares the extracted δ*n* with that expected from thermal excitations at the neutrality point. Above 10 K, δ*n* follows the parabolic dependence *n*_th_(*T*)/2 ∝ *T*^2^ (within measurement accuracy), matching the results from the broadening of the *ρ*_*xx*_(*n*) peak shown in Fig. 1c.

### Metal–insulator transition

**Extended Data Fig. 4:**
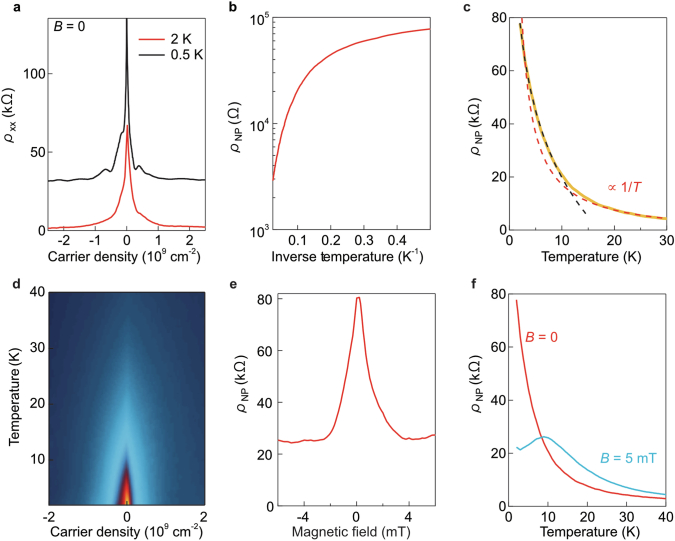
Insulating behavior at the neutrality point. **a** Zero-field *ρ*_xx_(*n*) at 0.5 and 2 K (color-coded curves offset by 30 kΩ for clarity). **b** Arrhenius plot of *ρ*_NP_(*T*). **c** Same data on linear scale (orange curve). Red dashed curve: Fit to *ρ*_NP_ ∝ 1/*T*, characteristic of ballistic Dirac-fermion transport in the Boltzmann regime. Black curve: Logarithmic fit below 10 K. **d** Evolution of *ρ*_NP_(*n*,*T*) near the NP (dark blue to yellow: 0 to 80 kΩ). **e** *ρ*_NP_ as a function of perpendicular *B* at 2 K, showing suppression of the insulating state by small magnetic fields. **f** Temperature dependence of *ρ*_NP_ at 0 and 5 mT (color-coded). The magnetoresistance changes sign around 10 K. Data for device S1 (*W* ≈ 9 μm).

At low *T*, our proximity-gated devices exhibited a highly resistive state at the neutrality point, which showed signatures of strong localization and quantum interference. Extended Data Fig. 4a is a plot of *ρ*_*xx*_(*n*) under zero *B*. There is a pronounced peak at the neutrality point, which reached approximately 100 kΩ at 0.5 K, that is, 5 times larger than expected for charge-neutral ballistic graphene in its minimum conductivity state^39^. This behaviour indicates a metal–insulator transition, probably of Anderson type, as previously observed in double-layer graphene heterostructures^40^. The resistivity overshoots the maximum metallic value^39^ of π*h*/4*e*^2^ ≈ 20 kΩ at *n* ≲ 10^8^ cm^−2^. This corresponds to the Fermi wavelength *λ*_F_ ≳ 3.5 µm, which becomes comparable to the device width *W*. In this regime, the number of electronic channels fitting *W* is reduced to less than 5, whereas the Fermi energy is only 10–15 K. Under these conditions, quantum confinement effects become significant and open energy gaps between quantized sub-bands, which can become comparable to *k*_B_*T*. The onset of spatial quantization could contribute to the observed insulating state, although Anderson-type localization is probably the dominant mechanism given the observed *B* and *T* dependences^40^. Away from the neutrality point, other ‘mesoscopic’ features appeared on the *ρ*_*xx*_(*n*) curves at subkelvin *T* (Extended Data Fig. 4a). Given the long *λ*_F_, these features can be attributed to interference (Fabry–Pérot) resonances caused by standing waves in our ballistic devices.

To elucidate the nature of the observed insulating state, we analysed the *T* dependence of resistivity at the neutrality point (*ρ*_NP_). Extended Data Fig. 4c shows an example of the measured *ρ*_NP_(*T*). The same data are replotted in Arrhenius coordinates in Extended Data Fig. 4b. The complete evolution of *ρ*_*xx*_(*n*) with *T* is presented in Extended Data Fig. 4d. These plots rule out the presence of simple thermal activation over an energy gap at the Dirac point, which would manifest as a straight line in the Arrhenius plot. Instead, *ρ*_NP_(*T*) has two distinct regimes: (1) logarithmically increasing with decreasing *T* below 10 K (black dashed curve in Extended Data Fig. 4c) and (2) a 1/*T* dependence at higher temperatures (red curve). The 1/*T* behaviour can be understood as follows. The density of thermally excited electrons and holes at the Dirac point is given by *n*_th_ ∝ *T*^2^ (Fig. 1c), whereas their effective masses *m*_th_ ∝ *T* (refs. ^18,25^). Consequently, the observed dependence *ρ*_NP_ ∝ 1/*T* implies a temperature-independent scattering time, *τ* = *m*_th_/(*e*^2^*n*_th_*ρ*_NP_), consistent with the ballistic transport of Dirac fermions limited by edge scattering and *τ* ≈ *W*/*v*_F_, as discussed in the main text.

The magnetic field dependence provides further insight into the metal–insulator transition. Extended Data Fig. 4e shows *ρ*_NP_(*B*) at 2 K. The insulating state was suppressed by fields of approximately 1 mT, resulting in pronounced negative magnetoresistance. We attribute this suppression to the breaking of time-reversal symmetry for interfering electron trajectories, a mechanism analogous to the destruction of weak localization in normal metals and which is expected to play a role in strong (Anderson-type) localization^18^. On increasing *T* above 10 K, the magnetoresistance changed sign from negative to positive, as evident from comparing *ρ*_NP_(*T*) at 0 and 5 mT in Extended Data Fig. 4f. This positive magnetoresistance is characteristic of a charge-neutral Dirac plasma in the Boltzmann regime^25^. The sign change occurs at approximately 10 K, providing independent confirmation of the regime change from the insulating state to the ballistic Dirac plasma, which was inferred above from changes in the functional form of *ρ*_NP_(*T*) (Extended Data Fig. 4c). The negative magnetoresistance also rules out an excitonic gap at the Dirac point. If present, such a gap would be enhanced by the magnetic field due to stronger confinement and increased exciton binding energy, contrary to our observations.

### Magnetic focusing in reference devices

**Extended Data Fig. 5:**
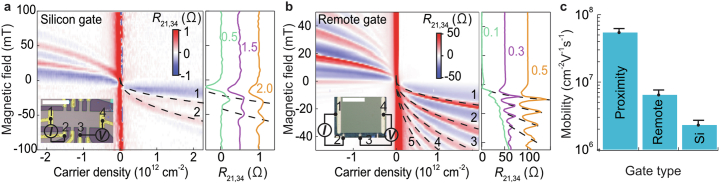
Magnetic focusing for different gate designs. **a** and **b** Measurements for reference devices with remote Si and graphite gates, respectively. Insets: Device micrographs and measurement configurations (current *I*_21_ between contacts 2 and 1; voltage *V*_34_ between 3 and 4; scale bars: 20 µm; *T* = 20 K). Color maps: Focusing resistance *R*_21,34_(*n*,*B*). Dashed curves indicate the expected position of magneto-focusing peaks (peak numbers *P* are labelled). Right panels: Vertical cuts from the maps at the labelled densities (in units of 10^12^ cm^−2^). **c** Transport mobilities found in our magneto-focusing experiments for devices with the three types of gates. The error bars indicate uncertainty in our evaluation of the extracted *μ*.

Extended Data Fig. 5a,b presents magneto-focusing measurements for hBN-encapsulated graphene devices with a Si-wafer gate (approximately 350 nm below the graphene) and a graphite gate (approximately 70 nm below), which are analogous to the measurements in Fig. 2 for proximity-gated devices. The focusing peaks appear at the expected positions given by^26,27^ *B*(*n*,*P*) = 2*ħ*(π*n*)^1/2^*P*/*eL* where *P* is the peak number and *L* the distance between injector and collector (dashed curves in Extended Data Fig. 5a,b).

We define the ‘first appearance’ of magneto-focusing peaks as the carrier density *n* where the *P* = 1 peak becomes clearly distinguishable above a noisy or fluctuating background, typically exceeding it by a factor of over 2. Importantly, the peak grows very rapidly as a function of *n*, so that the exact threshold factor is relatively unimportant and, in our experience, led to variations of less than 30% in the estimated onset density. Using this criterion, the magneto-focusing peaks in Extended Data Fig. 5a,b first appeared at *n* ≈ 0.5 and approximately 0.1 × 10^12^ cm^−2^ for the devices with Si and remote graphite gates, respectively. For the measurement geometries shown in the figure insets with injector–collector separations *L* ≈ 19 and 22.5 μm, this yielded transport mobilities *µ* = (*eℓ*/*ħ*)(π*n*)^−1/2^ ≈ 2 and 7 × 10^6^ cm^2^ V^−1^ s^−1^, respectively. These values are in good agreement with the mobilities extracted from the *ρ*_*xx*_(*n*) dependences of the devices at the same densities, which validates the criterion suggested for estimating transport mobilities from the appearance of the first peak in magneto-focusing measurements. Accordingly, we have used this criterion to estimate *µ* also in proximity-gated devices. The surprising accuracy of this rule-of-thumb criterion stems from the very rapid development of magneto-focusing signals with increasing *n*, which resembles the similarly simple criterion for estimating the quantum mobility as discussed in ‘Determination of quantum mobility’ in Methods.

Extended Data Fig. 5c summarizes our findings for mobility in all three types of device. Remote graphite gates improve *µ* several fold compared to Si-gated devices (which explains why graphite gates have become common in the recent literature), whereas proximity screening provides another order-of-magnitude enhancement in electronic quality. Note that the achieved mobilities in Extended Data Fig. 5c have to be compared at different carrier densities because magnetic focusing became observable only at *n* that exceeded δ*n* by 30–100 times, with δ*n* varying significantly between device types.

### Onset of SdH oscillations

**Extended Data Fig. 6:**
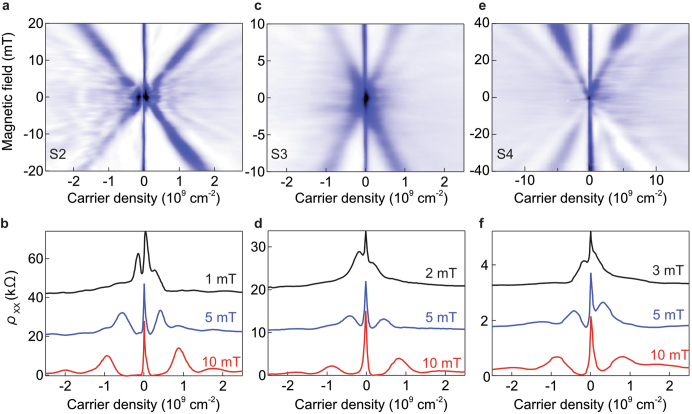
Onset of SdH oscillations in different proximity-gated devices. **a**, **c**, **e** Maps *ρ*_xx_(*n*,*B*) near the NP for devices S2, S3 and S4 (white-to-blue scales: 0 to 40, 25 and 3 kΩ, respectively). **b**, **d**, **f** Horizontal cuts from the maps above. All measurements at ∼2 K. Note that the early SdH oscillations appear superimposed on the pronounced resistivity peak at the NP, creating a broad dark region below a few mT in the color maps **a**, **c**, **e** that obscures the onset of Landau quantization. The onset becomes apparent at lower fields using horizontal cuts as in **b**, **d**, **f**.

We studied seven graphene devices with proximity gates, and results for three of them with remnant δ*n* < 10^8^ cm^−2^ are presented below. Extended Data Fig. 6 shows their Landau fan diagrams near the neutrality point, along with horizontal cuts at several fields in the millitesla range. Quantization becomes clearly visible below 1, 2 and 3 mT for devices S2, S3 and S4, respectively, so that *µ*_q_ exceeded 10, 5 and 3.3 × 10^6^ cm^2^ V^−1^ s^−1^, respectively. The quantum mobility in device S2 matched or exceeded that of device S1, as described in the main text. To ensure reliable identification of SdH oscillations, we considered them to emerge only if the resistivity minima at *ν* = ±2 became pronounced enough to rule out any possible quantum-interference features (main text and ‘Metal–insulator transition’ in Methods). This required fields notably higher than those needed for the criterion *µ*_q_B* ≈ 1, where SdH oscillations with amplitude less than exp(−π) ≈ 4% of total *ρ*_*xx*_ appear at B* (‘Determination of quantum mobility’ in Methods). Accordingly, the above values provide only lower bounds for *µ*_q_.

### Determination of quantum mobility

**Extended Data Fig. 7:**
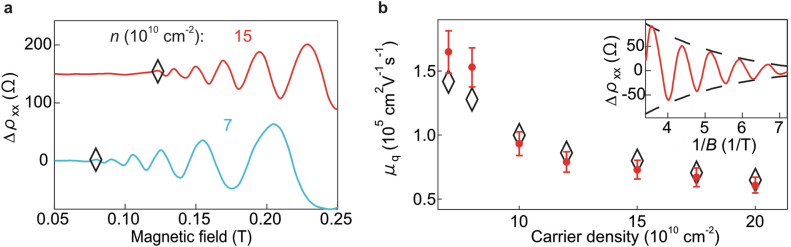
Analysis of SdH oscillations’ onset. **a** Oscillatory part of resistivity Δ*ρ*_xx_ for two representative densities (smooth background subtracted; curves are offset for clarity). Empty diamonds mark the first visible oscillations. **b** Quantum mobilities extracted using the rule-of-thumb criterion for the onset of SdH oscillations (diamonds) and using Lifshitz-Kosevich theory fits (red symbols). Inset: Data from **a** replotted as a function of 1/*B*. Dashed curves show Lifshitz-Kosevich fits through resistance maxima and minima.

Although the expression *µ*_q_B* ≈ 1 is widely adopted in the literature, to justify its use in our study, we performed a quantitative analysis of SdH oscillations. To avoid complications arising from the metal–insulator transition, QHE and interference resonances, the measurements were carried out on devices with remote graphite gates at an elevated *T* of 5 K. Under these conditions, the SdH oscillations exhibited clear sinusoidal behaviour, with their amplitude exponentially increasing with *B* (examples are shown in Extended Data Fig. 7a).

To analyse such curves, we applied the Lifshitz–Kosevich formula^41^

$$\Delta {\rho }_{xx}(n,T,B)\propto \frac{2{{\rm{\pi }}}^{2}{k}_{{\rm{B}}}T/\hbar {\omega }_{{\rm{c}}}}{\sinh (2{{\rm{\pi }}}^{2}{k}_{{\rm{B}}}T/\hbar {\omega }_{{\rm{c}}})}\exp (-{\rm{\pi }}/{\mu }_{{\rm{q}}}B),$$

where *ω*_c_ is the cyclotron frequency. For each carrier density, we replotted the oscillatory part of the resistivity Δ*ρ*_*xx*_ as a function of 1/*B* and fitted it using the Lifshitz–Kosevich expression, with *µ*_q_ as the only fitting parameter (inset of Extended Data Fig. 7b). Extended Data Fig. 7b compares the quantum mobilities extracted from these fits with those obtained using the rule-of-thumb criterion *µ*_q_B* ≈ 1 with B* being the field at which SdH oscillations first become clearly discernible (empty diamonds in Extended Data Fig. 7a). Within our experimental accuracy (10–20%), the two methods yield identical values of *µ*_q_, validating our use of the simpler criterion.

### Onset of quantized Hall plateaux

**Extended Data Fig. 8:**
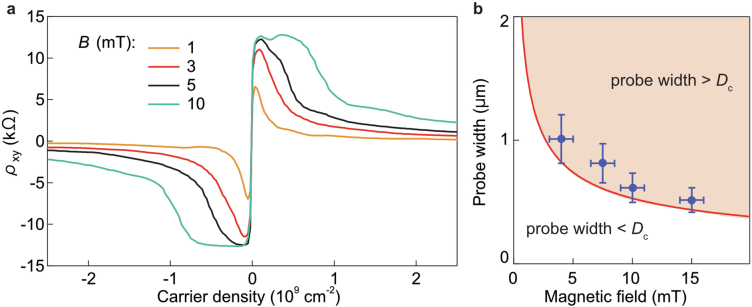
Contact width and Hall quantization. **a** Hall resistivity *ρ*_xy_(*n*) at different *B* (color-coded curves) for device S1. Hall plateaus emerge below 5 mT. **b** Magnetic field required for their emergence versus voltage probes’ width (blue symbols). Error bars indicate uncertainties in determining *w* (from optical micrographs) and the quantization onset. Red curve: Criterion *D*_c_ = *w*. The pink area shows where *w* exceeds *D*_c_, allowing entry of edge states into ohmic contacts.

Although in our proximity-gated devices quantum Hall plateaux appeared in fields as small as several millitesla, we found that contact geometry, rather than disorder, limited QHE onset. Extended Data Fig. 8a shows the Hall resistivity *ρ*_*xy*_(*n*) at different magnetic fields. As *B* was increased, the peaks of *ρ*_*xy*_ grew until they reached the quantized value of *h*/2*e*^2^ at 4–5 mT. At these fields, the cyclotron diameter *D*_c_ was comparable to the width *w* of our voltage probes (for Dirac fermions at *ν* = ± 2, *D*_c_ = 2*ℓ*_B_ ≈ 1.6 µm/*B*(mT)^1/2^). Consequently, cyclotron orbits at lower *B* were reflected, preventing edge states from reaching the contact regions and, thus, destroying the quantization^29^.

To corroborate this geometric effect, we examined QHE onset in devices with different *w*. Extended Data Fig. 8b plots the onset field versus *w* (blue symbols). Hall plateaux developed systematically at lower *B* for wider contacts, following qualitatively the criterion *D*_c_ ≲ *w*. This indicates that, in our proximity-gated devices, the onset of full quantization was still limited by the dimensions of the voltage probe rather than the homogeneity of graphene or mobility, and we expect the QHE in proximity-gated devices to fully develop in fields as low as 1 mT, if we use contacts wider than 2 μm.

### Fractional QHE under proximity screening

**Extended Data Fig. 9:**
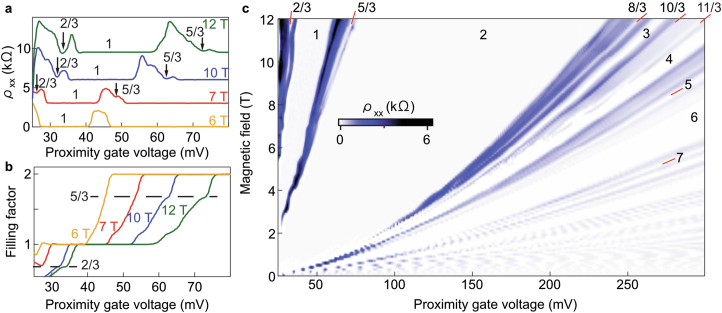
Evolution of fractional quantum Hall states with magnetic field. **a** and **b** *ρ*_xx_ and *ν* = (*h*/*e*^2^)/*ρ*_xy_ as functions of proximity gate voltage at different *B* from 6 to 12 T. Vertical arrows indicate minima in *ρ*_xx_. Horizontal dashed lines mark emerging Hall plateaus for *ν* = 2/3 and 5/3. **c** Map of *ρ*_xx_ as a function of *B* and proximity gate voltage. Numbers indicate different filling factors with red lines serving as guides to the eye.

To assess how the fractional QHE develops in our proximity-gated devices, we studied in detail its evolution with increasing *B*, as shown in Extended Data Fig. 9. No fractional states could be observed below 6 T. Their signatures at *ν* = 2/3 and 5/3 first appeared above 7 T and became more pronounced at higher fields (Extended Data Fig. 9a,b). Notably, no signatures of *ν* = 1/3 were observed in any of our devices, which we attribute to the large negative quantum capacitance, which increases the total capacitance near incompressible states and can lead to a complete collapse of visible gaps when measurements are performed as a function of gate voltage^31^.

The emergence of fractional states corresponds to the magnetic length *ℓ*_B_ decreasing below 10 nm. This is consistent with the fact that *ℓ*_B_ represents the spatial scale of electron–electron interactions responsible for the fractional QHE. This critical scale of 10 nm inferred from the emergence of fractional states at 7 T aligns well with the characteristic scale 2π*αd* ≈ 10 nm at which proximity screening for *d* ≈ 1 nm becomes effective in suppressing electron–hole puddles, as discussed in the main text. The systematic appearance of fractional QHE states only when *ℓ*_B_ < 10 nm confirms that proximity screening primarily affects long-range interactions while preserving the short-range ones essential for many-body phenomena in high magnetic fields.

### Sources of disorder

Despite considerable progress in improving the quality of graphene devices over the past decade, the primary sources of disorder limiting mobility and charge homogeneity in hBN-encapsulated graphene remain poorly understood. Our current experiments and the existing literature allow us to rule out several potential disorder sources. First, atomic-scale defects in the graphene itself or at graphene/hBN interfaces are unlikely to be the dominant scatterers. Scanning probe experiments would have revealed individual short-range defects that were clearly visible on the corresponding images^42^ and did not occur within areas as large as over 10 µm^2^. Graphene/hBN interfaces are known to be atomically clean and flat, with minimal interfacial contamination^43^ (except for bubbles and wrinkles, which, however, were absent in our devices; Supplementary Information). Second, charged impurities at the metal/hBN interface of the top gate also appear to play a minimal role. Further experiments demonstrated no significant improvement when using graphite for both top and bottom gates (placed at distances of more than 10 nm). The latter observation is in line with previous studies using double-gated devices with two graphite gates, which found that mobilities were comparable to those in our remote-graphite-gated devices. Furthermore, attempts to use other atomically flat metallic crystals as proximity gates (including Bi_2_Sr_2_CaCu_2_O_8__+*x*_ and TaS_2_) failed to yield high mobilities^21^, presumably due to charged impurities in contact with hBN at their surfaces. Third, elastic strain in graphene can generate electron–hole puddles, but our observations indicate this to be an improbable primary source of disorder. Indeed, strain-induced puddles cannot be suppressed by proximity gating, yet we observed a notable reduction in the charge inhomogeneity.

Beyond edge scattering, which obviously dominates in our limited-width devices, charged impurities within hBN represent the most probable source of both residual disorder and charge inhomogeneity. The hBN crystals used in our devices (from the National Institute for Materials Science) contain impurities at concentrations of approximately 10^15^ cm^−3^, primarily carbon substituents (T. Taniguchi, private communication). In comparison, state-of-the-art GaAlAs heterostructures^8,9^ contain impurities at concentrations of less than 5 × 10^13^ cm^−3^. However, this comparison requires nuanced interpretation. Impurities in hBN typically lie deep within the bandgap and remain predominantly neutral, whereas impurities in GaAlAs heterostructures (such as Si used for doping) are mostly ionized. Nonetheless, it is plausible that a fraction of deep impurity states in hBN become charged, creating a background electrostatic potential in graphene. The observed effectiveness of proximity gating (particularly in suppressing charge inhomogeneity by two orders of magnitude) strongly supports this interpretation. Proximity screening specifically targets long-range electrostatic disorder while having a minimal impact on short-range scattering mechanisms. Although this analysis provides useful insights, a definitive conclusion about the dominant source(s) of disorder in hBN-encapsulated graphene demands further systematic investigation. This will probably require larger devices, as the mobilities achieved in our proximity-gated devices have been limited by edge scattering due to their finite size.

### Helical quantum Hall transport

**Extended Data Fig. 10:**
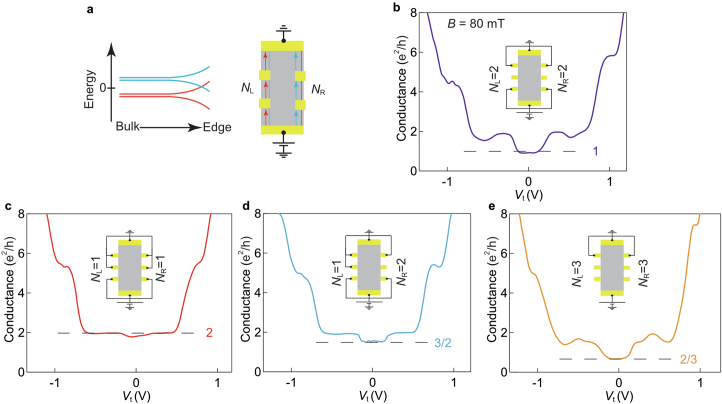
Helical quantum Hall transport. **a**, Left: Schematic of the zeroth Landau level in the ferromagnetic phase leading to helical edge states. Colors indicate spin-up and spin-down valley-degenerate Landau level pairs (red and blue, respectively). Right: Two-terminal measurement schematics. Yellow rectangles represent ohmic contacts. Arrows along edges indicate helical edge state propagation directions. *N*_L_ and *N*_R_ denote the numbers of helical-conductor sections at the left and right edges, respectively. **b**-**e** Two-terminal conductance *G*_2t_ as a function of top gate voltage *V*_t_ at 80 mT for different measurement configurations: *N*_L_ = *N*_R_ = 2 (**b**), *N*_L_ = *N*_R_ = 1 (**c**), *N*_L_ = 1 and *N*_R_ = 2 (**d**), and *N*_L_ = *N*_R_ = 3 (**e**). Dashed lines indicate the expected conductance values near *V*_t_ = 0 for each geometry. All measurements at 2 K.

Although proximity screening suppresses electron–electron interactions, it can enable quantum phases that are otherwise difficult to observe in remote-gate devices. A notable example is the helical quantum Hall phase in the zeroth Landau level, which arises if a spin-polarized ferromagnetic phase is stabilized either by very large in-plane magnetic fields (approximately 30 T)^44^ or by screening Coulomb interactions using high-*ε* substrates^35^. In standard graphene devices, the zeroth Landau level typically exhibits an antiferromagnetic ground state, resulting in an insulating bulk and insulating edges. By contrast, the ferromagnetic phase leads to a quantum Hall topological insulator with an insulating bulk but conducting helical edge channels where the current propagation direction is locked to electron spin polarization^35,44^ (Extended Data Fig. 10a).

To test for helical edge transport in proximity-gated graphene, we measured the two-terminal conductance in different contact configurations under a perpendicular magnetic field. In our multi-terminal Hall bar geometry, each ohmic contact acts as an equilibration reservoir for counter-propagating edge states, and therefore, each edge section between two contacts represents an ideal helical quantum conductor with resistance *h*/*e*^2^. Considering conduction along both device edges, the two-terminal conductance can be written as *G*_2t_ = (*e*^2^/*h*)(*N*_R_^−1^ + *N*_L_^−1^), where *N*_R_ and *N*_L_ are the numbers of helical-conductor sections for the right and left edges, respectively (Extended Data Fig. 10a, right panel).

Extended Data Fig. 10b shows the measured two-terminal conductance versus top gate voltage *V*_t_ at *B* = 80 mT, a relatively small but sufficient field for suppressing the insulating state at the neutrality point (‘Metal–insulator transition’ in Methods). As expected, well-defined conductance plateaux appeared at 2*e*^2^/*h* and 6*e*^2^/*h*, corresponding to the filling factors *ν* = ±2 and *ν* = ±6, respectively. Additionally, a quantized plateau emerged at the neutrality point (*V*_t_ = 0) with *G*_2t_ = *e*^2^/*h*, consistent with helical edge conduction and matching the expected value for our geometry with *N*_L_ = *N*_R_ = 2. Geometry-dependent measurements (Extended Data Fig. 10c–e) further support the presence of helical transport, as altering the number of contacts along the edges between the source and drain changed the conductance, as expected from the above formula. In all configurations, the observed conductance at the neutrality point showed quantized plateaux matching theoretical predictions.

These results demonstrate helical edge states in proximity-screened graphene at magnetic fields more than an order of magnitude lower than previously required (more than 1 T) using a high-*ε* substrate^35^. This highlights the potential of proximity-gated devices for accessing exotic quantum Hall phases at reduced magnetic fields by suppressing electron interactions.

## Online content

Any methods, additional references, Nature Portfolio reporting summaries, source data, extended data, supplementary information, acknowledgements, peer review information; details of author contributions and competing interests; and statements of data and code availability are available at 10.1038/s41586-025-09386-0.

## Supplementary information

Supplementary Information This file contains the Supplementary Methods and Supplementary Fig. 1.

## Data Availability

The original data files that support the findings of this study are available at Zenodo (10.5281/zenodo.15786631)^45^ and from D.D.
