# Supplementary material for: Proximity screening greatly enhances electronic quality of graphene
Source: Nature. 2025 Aug 20;644(8077):646–51. doi: 10.1038/s41586-025-09386-0 (PMC12367531; doi:10.1038/s41586-025-09386-0)
Supplement: Supplementary file 1 — This file contains the Supplementary Methods and Supplementary Fig. 1. [file 41586_2025_9386_MOESM1_ESM.pdf]

nature portfolio

https://doi.org/10.1038/s41586-025-09386-0

Supplementary information

Proximity screening greatly enhances electronic quality of graphene

In the format provided by the authors and unedited

Supplementary Methods

Graphene and hBN exfoliation

The hBN and graphene crystals were obtained via mechanical exfoliation of bulk crystals using 'blue tape' ELP BT 130E-SL from *Nitto Denko*. The key steps in the exfoliation process are as follows:

1. Oxidized silicon substrates were cleaned by ultrasonic treatment in acetone for 5 minutes, followed by isopropyl alcohol (IPA) for another 5 minutes. After drying with nitrogen (N_2), the substrates were baked on a hotplate at 150 °C.
2. Graphite crystals (*NGS Naturgraphit*) were applied to the blue tape and cleaved 5–7 times, which optimized surface coverage while maintaining large lateral dimensions of individual crystals.
3. The tape with exfoliated thin graphite crystals was applied to the silicon substrate immediately after the substrate was removed from the hotplate. To further enhance exfoliation yield, we sometimes pretreated substrates with low-power O_2 plasma before exfoliation.
4. For hBN exfoliation, bulk crystals (National Institute for Materials Science, Tsukuba, Japan) were cleaved 5–8 times before applying the blue tape. To improve the exfoliation yield, the tape was pressed onto and then peeled from the substrate at 80 °C. Over the course of our experiments, we utilized several different batches of hBN crystals and found that the specific batch had no noticeable influence on the electronic quality of our proximity-gated devices.
5. To facilitate identification of ultrathin hBN flakes (~1 nm thick), we performed the exfoliation on Si wafers with a 70 nm SiO_2 layer.
6. Exfoliation and assembly were done in air. Exfoliated graphene and hBN crystals were either used as soon as possible (typically within one hour) or kept in a glove box for later usage within maximum 2-3 weeks.

Assembly using PDMS stamps

For proximity-gated devices and some of the reference devices, heterostructures were assembled using polydimethylsiloxane (PDMS) stamps coated with a layer of polypropylene carbonate (PPC). PDMS sheets (*Gel-Pak*, DGL-45X45-0170-X4, 1.7 mm thick) were cut into 5 mm × 5 mm squares, affixed to plasma-cleaned glass slides, and spin-coated with a PPC solution (1 g PPC in 10 mL chloroform) at 3,000 rpm. The prepared stamps were then annealed in air at 130 °C for 5 minutes before use in assembly procedures.

The assembly began with picking up the top hBN crystal, followed sequentially by graphene and the bottom hBN crystal, with the temperature held at 45 °C throughout all steps. The resulting trilayer stack was then deposited onto a graphite crystal (or a clean oxidized Si wafer for some reference devices) at 120 °C, which melted the PPC layer. PPC residues were removed using acetone, and the assembled heterostructure was subsequently annealed in vacuum at 250 °C for 2 hours for further cleaning.

To minimize the formation of hydrocarbon-filled bubbles and interfacial wrinkles during the assembly, we emphasize that 2D crystals should be picked up from substrates and deposited onto the target layer/substrate as slowly as possible. This slower transfer allowed contaminants to be driven outside the atomically-flat interface between the two assembled crystals. Supplementary Fig. 1 shows images of our devices with large areas free from bubbles and wrinkles, which enabled the fabrication of final extra-large devices.

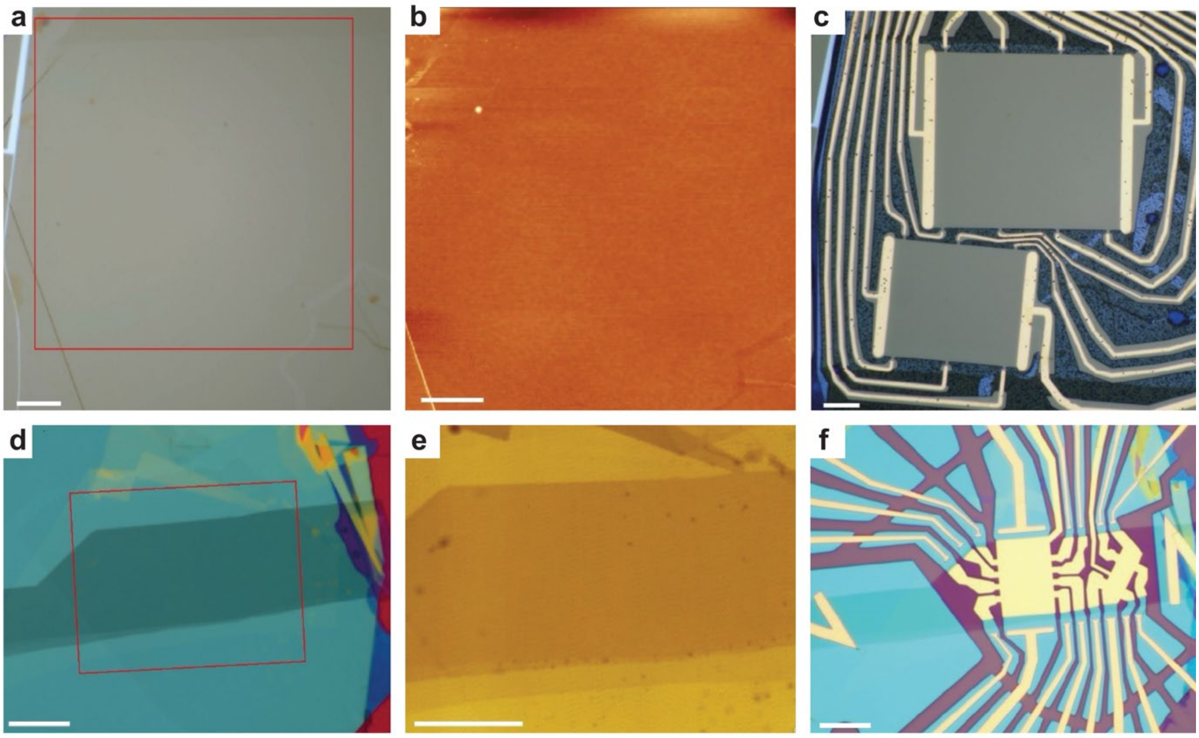

Supplementary Fig. 1| Optical micrographs of hBN-encapsulated graphene stacks used in devices with remote graphite gates (**a**) and with proximity gates (**d**), shown before microfabrication. Regions of interest are indicated by red boxes, with their AFM topography shown in panels **b** and **e**, whereas images of the final devices are shown in **c** and **f**, respectively. Scale bars, 10 µm for all panels.

Assembly using silicon nitride membranes

For some of our reference devices with remote graphite gates, we fabricated heterostructure stacks using polymer-free silicon nitride cantilevers. The assembly technique has been described previously[17] in extensive detail, including both the assembly process and cantilever fabrication. Here we provide further details and parameters specific to the heterostructure devices used in the present work.

Fresh silicon-nitride cantilevers were coated with a trilayer metal stack consisting of 1 nm of tantalum (Ta) for adhesion, 5 nm of platinum (Pt) to provide a smooth surface, and 0.75 nm of gold (Au) to promote adhesion with 2D materials. The top hBN crystal was picked up at 150 °C, followed sequentially by graphene and a bottom hBN layer (up to 70 nm thick) at 100 °C. The fully assembled hBN–graphene–hBN heterostructure was then released onto a large graphite flake at temperatures above 200 °C.

The entire assembly process was performed relatively rapidly, as elevated temperatures significantly enhanced the surface mobility of contaminant molecules, making the time of contact formation between 2D crystals less critical for avoiding contamination bubbles.

To ensure reliable release of the assembled stack from the cantilever, a substrate with high interfacial adhesion was necessary. Typically, thick (~50 nm) and laterally large (>100 µm) graphite crystals were used for this purpose, which also later served as remote gates. If graphite flakes were much thinner or smaller than heterostructure stacks, there was a high risk of failed release.

Spectroscopic and microscopic characterization of heterostructures

We employed Raman spectroscopy to verify the presence and determine thicknesses of thin hBN crystals as well as to confirm monolayer graphene crystals. Atomic force microscopy (AFM) provided surface topography, enabling identification of regions free from interfacial bubbles, wrinkles, and cracks as shown in Supplementary Fig. 1b,e. Additionally, AFM was used to measure the thickness of individual layers within each stack.

Microfabrication

Following heterostructure assembly, electron-beam lithography was used to define the top gate region, followed by deposition of 2 nm Cr/50 nm Au using electron-beam evaporation. In a subsequent lithography step, electrical contacts were fabricated by first exposing graphene edges (using reactive-ion etching with a 90% CHF_3 – 10% O_2 gas mixture) and then depositing 2 nm Cr / 70 nm Au to form one-dimensional edge contacts. For the final step, the metallic top gate and additional lithographically defined links served as an etching mask to define multiterminal Hall bars, as shown in the main text and Supplementary Fig. 1c,d.
